# Impact of new electrocardiographic criteria in arrhythmogenic cardiomyopathy

**DOI:** 10.3389/fphys.2012.00352

**Published:** 2012-09-17

**Authors:** Richard N. W. Hauer, Moniek G. P. J. Cox, Judith A. Groeneweg

**Affiliations:** ^1^Department of Cardiology, University Medical Center UtrechtUtrecht, Netherlands; ^2^Interuniversity Cardiology Institute of the NetherlandsUtrecht, Netherlands

**Keywords:** electrocardiography, diagnosis, ventricular tachycardia, cardiomyopathy, arrhythmogenic right ventricular dysplasia

## Abstract

Arrhythmogenic cardiomyopathy (AC) has originally been described as a disorder characterized by fibrofatty replacement of the myocardium, primarily of the right ventricle (RV), and ventricular tachyarrhythmias, sudden death, and at a late stage progressive heart failure. Arrhythmogenic right ventricular dysplasia or cardiomyopathy (ARVD/C) was the previous name of the disease. However, similar histopathologic changes are also found in the left ventricle (LV). AC is also considered a hereditary disease. Recent molecular genetic studies provide accumulating evidence that fibrofatty replacement is preceded by mutation-related desmosomal changes. Desmosomal dysfunction may lead to mechanical and thereafter electrical uncoupling, ultimately resulting in conduction delay. This activation delay and conduction block, provide a substrate for re-entrant mechanisms and thereby ventricular tachycardia (VT). The gold standard for AC diagnosis is demonstration of transmural fibrofatty replacement in cardiac tissue obtained by autopsy or surgery. To facilitate diagnosis in clinical practice, an international Task Force defined in 1994 a set of criteria (TFC) based on electrocardiographic, functional and morphologic features, and family history. These criteria have recently been revised. Routine 12-lead electrocardiography is one of the most important tools for AC diagnosis in all stages of the disease. Even in the absence of other markers in the early concealed stage of the disease, in line with early slow conduction and electrical uncoupling ECG analysis may contribute to early diagnosis. Activation delay and site of origin of VT are reflected in various characteristics of the surface 12-lead electrocardiogram. Since the ECG is easy to obtain, this technique is particularly useful, for both diagnosis and follow up of disease progression.

## Introduction

Arrhythmogenic cardiomyopathy (AC) has originally been described as a disorder histopathologically characterized by fibrofatty replacement of the myocardium, primarily of the right ventricle (RV), and clinically by ventricular tachyarrhythmias, sudden death, and at a late stage progressive heart failure (Marcus et al., 1982; Basso et al., 1996, 2009; Roguin et al., 2004; Dalal et al., 2005; Piccini et al., 2005; Cox et al., 2008). Arrhythmogenic right ventricular dysplasia or cardiomyopathy (ARVD/C) was the previous name of the disease. However, similar histopathologic changes are also found in the left ventricle (LV). Moreover, at the molecular level both ventricles and also the interventricular septum are equally affected by down-regulation and altered distribution of intercalated disk proteins. These observations made AC the preferred terminology.

AC is also considered a hereditary disease. Recent molecular genetic studies provide accumulating evidence that fibrofatty replacement is preceded by mutation-related desmosomal changes (McKoy et al., 2000; Protonotarios et al., 2001; Rampazzo et al., 2002; Gerull et al., 2004; Dalal et al., 2006; Pilichou et al., 2006; Syrris et al., 2006a,b; den Haan et al., 2009; Cox et al., 2011). Desmosomes are protein complexes located in the intercalated disk between adjacent cells and are crucial for maintaining mechanical coupling of the cardiomyocytes. Several reports have shown that alterations in one or multiple desmosomal proteins affect expression and distribution of other desmosomal and other non-desmosomal intercalated disk proteins, such as Connexin43 and the sodium channel Nav1.5, responsible for electrical coupling and conduction, respectively (Oxford et al., 2007; Sato et al., 2009). In this way mechanical uncoupling gives rise to electrical uncoupling and slow conduction (Kaplan et al., 2004a,b; Oxford et al., 2007; Asimaki et al., 2009; Noorman et al., 2009; Sato et al., 2009).

The relationship between this mechanical and electrical uncoupling and fibrofatty replacement is largely unknown. However, the hypothesis that in AC patients cardiac cellular uncoupling precedes fibrofatty alteration is strongly supported by identification of an altered distribution of desmosomal proteins and Connexin43, in histologically still unaffected left ventricular and septal tissue (Asimaki et al., 2009). This observation may have diagnostic implications in the early concealed stage of the disease, characterized by still absent or minor histopathological tissue alteration. However, sudden death may occur at that stage as first manifestation of AC (Thiene et al., 1988; Corrado et al., 1997). Slow conduction and electrical uncoupling and at a later stage altered tissue architecture due to the fibrofatty infiltration, lead to inhomogeneous activation delay by electrical conduction block, lengthening of conduction pathways, and load mismatch at pivotal points. This activation delay and conduction block, provide a substrate for re-entrant mechanisms and thereby ventricular tachycardia (VT) (Spear et al., 1979; de Bakker et al., 1993; Cabo et al., 1994; Fast and Kléber, 1997; Kaplan et al., 2004a,b). Previous invasive electrophysiologic studies have confirmed that VT in patients with AC is due to reentry (Ellison et al., 1998; Marchlinski et al., 2004).

Why fibrofatty alteration is usually more prominent in the RV is still unclear. A larger stretch at the thin RV wall has been suggested as a potentially causative factor (Basso et al., 2009). A histologically dominant RV involvement seems to be related to a frequently observed right ventricular origin of monomorphic VT, showing left bundle branch block (LBBB) morphology. Studies on the origin of polymorphic VT and ventricular fibrillation are lacking. Both ventricles are not homogeneously affected. Marcus et al. described already in 1982 the so-called “triangle of dysplasia,” being the RV outflow tract, an area below the tricuspid valve, and the RV apex (Marcus et al., 1982). However, other areas in the RV, as well as the LV may be affected. Histologically and in imaging studies septal involvement is not common in AC. However, septal fibrosis is frequently found with cardiac sarcoidosis. Clinically, cardiac sarcoidosis can mimic the AC phenotype as well (Ladyjanskaia et al., 2010). The differential diagnosis of AC versus cardiac sarcoidosis is crucial since management in both diseases is very different (Ladyjanskaia et al., 2010).

The gold standard for AC diagnosis is demonstration of transmural fibrofatty replacement in cardiac tissue obtained by autopsy or surgery. To facilitate diagnosis in clinical practice, an international Task Force defined in 1994 a set of criteria (TFC) based on electrocardiographic, functional and morphologic features, and family history (McKenna et al., 1994). Data of growing numbers of index cases and their family members, combined with molecular genetic data, increased insight in development and behavior of the disease process importantly (Hulot et al., 2004; Dalal et al., 2006; Cox et al., 2011). Therefore, recently a new Task Force introduced modifications to the 1994 TFC by implementation of these new insights (Marcus et al., 2010). Similar as in the 1994 TFC, also in the new TFC abnormalities were subdivided into major and minor according to the specificity for AC. AC diagnosis was based on the combination of either two major criteria, or one major and two minor, or four minor criteria. Criteria were derived from: (1) global or regional dysfunction and structural alterations, (2) tissue characterization, (3) depolarization abnormalities, (4) repolarization abnormalities, (5) arrhythmias, and (6) family history.

Routine 12-lead electrocardiography is one of the most important tools for AC diagnosis in all stages of the disease. Even in the absence of other markers in the early concealed stage of the disease, in line with early slow conduction and electrical uncoupling ECG analysis may contribute to early diagnosis.

Activation delay and site of origin of VT are reflected in various characteristics of the surface 12-lead electrocardiogram. Since the ECG is easy to obtain, this technique is particularly useful, not only for AC diagnosis, but also for evaluation of disease progression during follow-up. In addition, 12-lead ECG recording of a specific VT morphology is crucial to select and map the VT for catheter ablation procedures.

## Twelve-lead ECG during normal sinus rhythm

### Activation delay (depolarization) parameters

**Figure 1:**
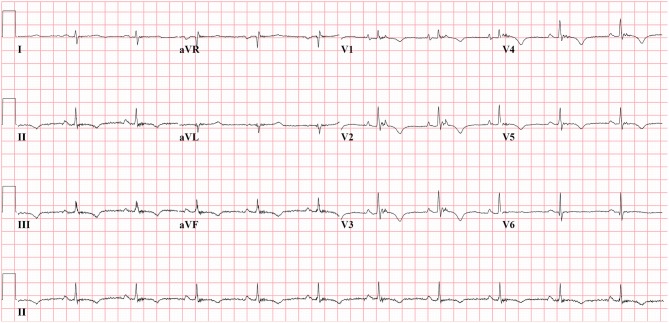
**Epsilon waves in precordial leads V_1−3_ from AC patient with plakophilin2 mutation.** Epsilon wave is a distinct deflection clearly separated from QRS complex. Epsilon wave remains a major criterion in the new Task Force criteria.

Activation delay due to cellular uncoupling and slow conduction, and altered tissue architecture by fibrofatty replacement is often visible in the ECG. In the original descriptions and 1994 TFC, typical manifestations are epsilon waves and widening of the QRS complex in leads V_1_–V_3_ (McKenna et al., 1994). Epsilon waves and localized QRS prolongation are major criteria of the 1994 TFC. An epsilon wave was defined as a distinct deflection after the end of the QRS complex, i.e., after the QRS complex had returned to the isoelectric line (Figure 1) (Fontaine et al., 1993). In the new TFC the epsilon wave remained as a major criterion, but the widening of the QRS complex was deleted, since discrimination from right bundle branch block (RBBB) may be difficult. Although epsilon waves are highly specific for AC, sensitivity is low. Cox et al. (Cox et al., 2008) observed an epsilon wave in only 4 out of 42 AC patients (10%). Because of this limited sensitivity, other parameters have been evaluated.

Peters et al. (Peters and Trummel, 2003) determined increased QRS ratio by using the QRS duration of all precordial leads in the formula (V_1_+V_2_+V_3_)/(V_4_+V_5_+V_6_) > 1.2 to solve the problem of discrimination with RBBB. However, also this criterion was found in only 35% of patients with AC (Cox et al., 2008). Nasir et al. reported the delayed S wave upstroke defined from the nadir of the S wave up to the isoelectric line in V_1−3_ ≥ 55 ms, as a sensitive criterion representing activation delay (Nasir et al., 2004).

**Figure 2:**
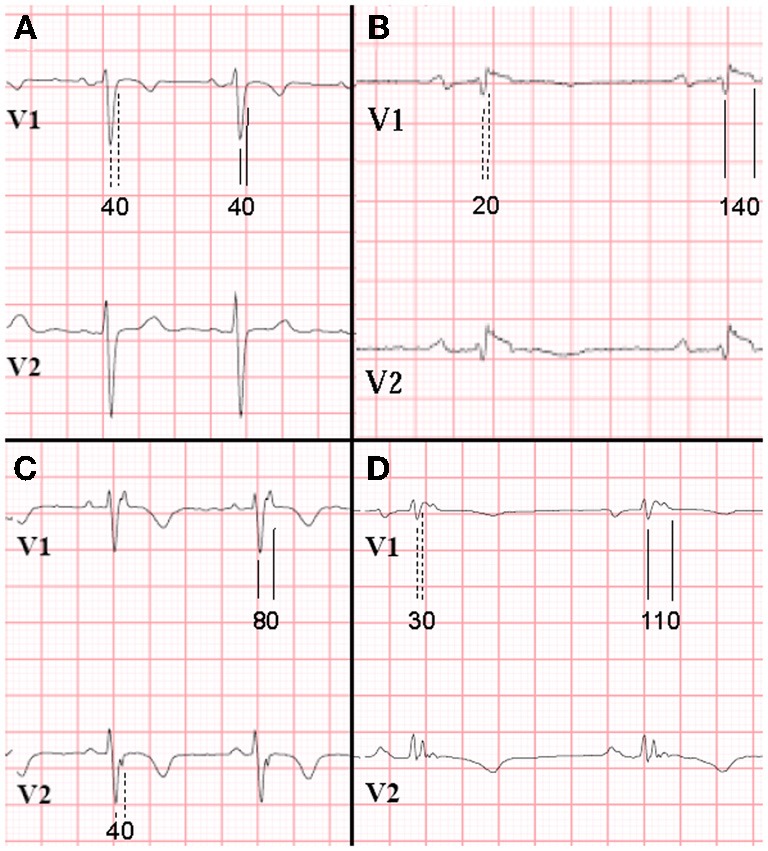
**ECG recordings V1 and V2 of 4 patients in sinus rhythm, while off drugs (25 mm/s, low-pass filter 100 Hz).** RBBB was absent (RBBB defined as QRS duration ≥120 ms in all leads). Difference in measurement results (in ms) of S wave upstroke (1st complexes, dashed lines) and terminal activation duration (TAD; 2nd complexes, continuous lines). Panel **A** (control patient) shows no differences in S wave upstroke and TAD, both being normal. On the contrary, in panels **B**–**D** (AC patients), S wave upstroke <55 ms, whereas TAD is prolonged. Prolonged TAD is a minor criterion in the new Task Force criteria. [Reprint with permission from Cox et al. (2008)].

**Figure 3:**
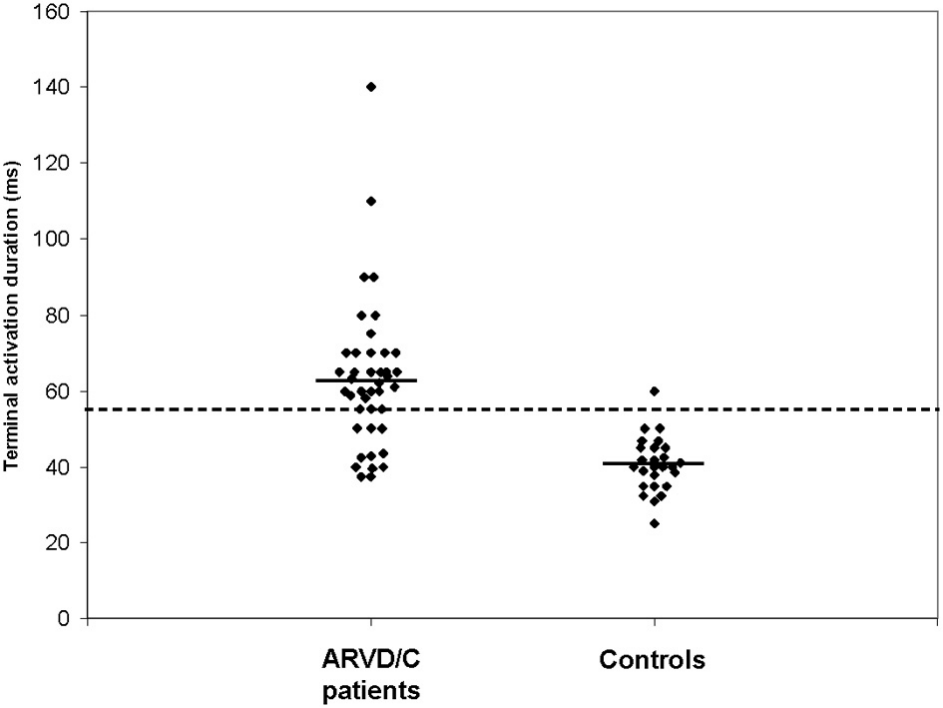
**Results of measurements of terminal activation duration (TAD) of AC patients and idiopathic VT controls.** Mean values are indicated by horizontal lines. AC patients had a significantly longer TAD than idiopathic VT control patients (*P* < 0.001). The dashed line indicates the cut-off point of 55 ms. [Reprint with permission from Cox et al. (2008)].

Our group introduced prolonged Terminal Activation Duration (TAD) (Cox et al., 2008). TAD is defined as the longest value in V_1−3_, from the nadir of the S wave to the end of all depolarization deflections, thereby including not only the S wave upstroke, but also both late fractionated signals and epsilon waves (Figure 2). Thus, total activation delay was conveyed by this new parameter. In Figure 2, the difference between S wave upstroke and TAD is clearly visible. TAD was considered prolonged if ≥55 ms, and only applicable in the absence of complete RBBB. The same value was applied as determined for prolonged S wave upstroke by Nasir et al. (2004) since it proved to be a cut-off point with high specificity also in our study. Prolonged TAD appeared to be the most sensitive activation delay criterion. It was recorded in 30 of 42 AC patients (71%). Prolonged TAD was not identified in 26 of 27 patients with idiopathic VT (Figure 3). Because of the superiority in sensitivity and the high specificity of prolonged TAD, this new criterion was included in the new 2010 TFC (Marcus et al., 2010). Cox et al. reported recording of prolonged TAD in 5 out of 16 young (age <20 years) relatives of a pathogenic mutation-carrying proband with AC. In four of them it was the only identified abnormality. These findings suggest that prolonged TAD is an important marker in the early identification of AC.

It has to be realized that these diagnostic criteria use only V_1−3_, facing the RV outflow tract. Therefore, all criteria mentioned might reflect activation delay primarily of this part of the RV. Other still undefined criteria are needed to reflect alterations in other parts of the right and also LV.

### Repolarization parameters

**Figure 4:**
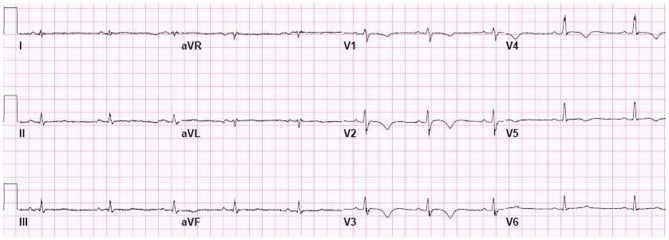
**Negative T waves in V_1−5_, recorded from AC patient with both a plakophilin2 mutation and a desmoglein2 mutation.** Negative T waves in V_1−3_ and beyond are a major criterion in the new Task Force criteria.

Abnormalities in repolarization in patients with AC are recorded as inverted T waves. In the 1994 TFC, inverted (negative) T waves in V_1−3_ or beyond were considered a minor criterion for AC diagnosis in the absence of RBBB and only if the patient was older than 12 years (Figures 1, 4). Because of the high specificity for AC, this criterion was upgraded to a major criterion in the new TFC, for individuals older than 14 years of age and in the absence of complete RBBB. In our series of 42 AC patients, this criterion was met in 28 patients (67%) and in none of the patients with idiopathic VT (Cox et al., 2008). Thus, sensitivity and specificity are similar to prolonged TAD.

In the new TFC, 3 additional criteria were included as minor criteria:

1. Inverted T waves *only* in leads V_1−2_ in individuals older than 14 years of age and in the absence of complete RBBB (Marcus et al., 2010). This criterion was identified in 4 of our 42 patients (10%) (Cox et al., 2008).
2. Inverted T waves only in V_4−6_.
3. Inverted T waves in leads V_1−4_ in individuals older than 14 years of age in the presence of RBBB (Marcus et al., 2010). This criterion was added since RBBB may be due to local activation delay and a negative T wave in V_4_ and beyond is very unlikely in classic RBBB.

## Twelve lead ECG during VT

### VT parameters for diagnosis and localization of site of origin

VT morphology and number of VT morphologies reflect location and extent of the disease process. In the absence of severe left ventricular and septal structural disease, a VT with LBBB morphology (dominant negativity in V_1_) means a site of origin in the RV. This is why AC is associated with monomorphic VT with LBBB morphology. In the 1994 TFC, recording of this VT morphology was a minor criterion for AC diagnosis (McKenna et al., 1994). However, we hypothesized that additional information could be obtained if the axis of the VT was taken into account.

**Figure 5:**
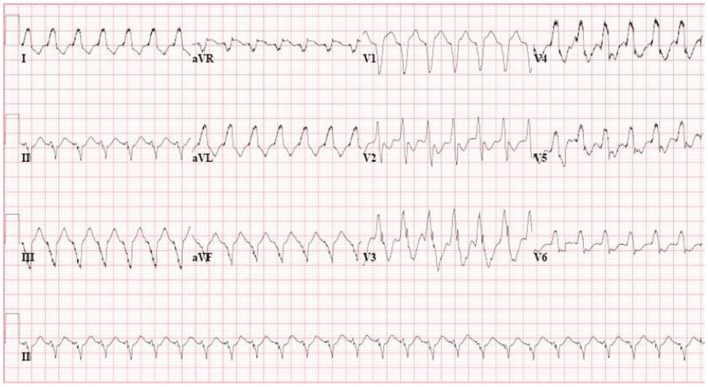
**VT with LBBB morphology and superior axis, recorded from AC patient with plakophilin2 mutation.** This morphology is a major criterion in the new Task Force criteria.

**Figure 6:**
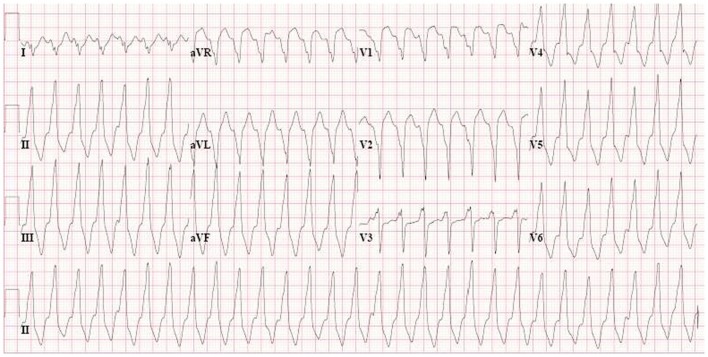
**VT with LBBB morphology and inferior axis (minor criterion), recorded from AC patient without identified desmosomal gene mutation.** This morphology is not typical for AC and is also often recorded in patients with idiopathic VT.

Idiopathic VT originating from the RV outflow tract, always shows LBBB morphology with an inferior axis. In contrast, in AC, affected areas are also found in other parts of the RV. Consequently, VT episodes originating from these areas can show LBBB morphology with a non-inferior axis as well. We evaluated the occurrence of LBBB VT with a superior axis arbitrarily defined from −30° to −150° (Figure 5) (Cox et al., 2008). This morphology was recorded in 27 of 42 patients (64%) with AC diagnosed according to the 1994 TFC. None of the 27 patients with idiopathic VT had this morphology. Thus, also this criterion had a similar specificity and sensitivity as prolonged TAD (≥55 ms) and negative T waves in V_1−3_. In accordance with our definition, recording of a VT with LBBB morphology and superior axis, defined as negative or indeterminate QRS in leads II, III, and aVF, and positive in lead aVL, became a major criterion in the new TFC (Marcus et al., 2010). A VT with LBBB morphology and inferior axis remained a minor criterion (Figure 6). The number of premature ventricular complexes on Holter monitoring required for counting as a minor criterion, decreased to 500 per 24 h.

Because of the variable extent of the disease process in AC, the number of VT morphologies may vary as well. Thus, multiple VT morphologies may be recorded in a single patient. Previous studies showed mean numbers of different VT morphologies per patient ranging from 1.8 to 3.8 (Ellison et al., 1998; O'Donnell et al., 2003). The number of different VTs in AC patients was quantified and compared to a control group after 8 years of follow-up (Cox et al., 2008). Multiple VT morphologies were recorded in 27 of 42 AC patients (64%), whereas the control group with idiopathic VT had only a single morphology. This study confirmed that occurrence of multiple VT morphologies is very common in AC patients. In case only a single VT morphology occurred spontaneously, programmed electrical stimulation (PES) contributed to yield multiple morphologies. In total, 10 additional AC patients or in total 88% fulfilled the multiple VT morphology criterion (Cox et al., 2008). Because of overlap with the superior axis criterion, the number of VT morphologies is not included in the new TFC.

## Validation of additional ECG criteria

Since sudden death frequently is the first manifestation of AC, even already in adolescence, the main diagnostic challenge is to identify individuals in an early concealed stage of the disease. We studied whether the previously proposed additional ECG criteria would improve diagnosis in patients highly suspected of AC, but *who do not fulfilled the 1994 TFC* for AC diagnosis (Cox et al., 2009). The presence of the new additional criteria was studied in so-called “probable” AC patients since they fulfilled either only 1 major and 1 minor, or only 3 minor criteria. We studied the diagnostic contribution of (1) prolonged TAD in V_1−3_, (2) VT with LBBB morphology and superior axis, and (3) recording of multiple VT morphologies. Two groups were studied: Group A (*n* = 21) with probable but not proven AC index patients, and Group B (*n* = 12) consisting of family members of 50 other proven (fulfilling 1994 TFC) AC index patients.

In Group A, none had epsilon waves or QRS >110 ms, whereas prolonged TAD was recorded in 7 (33%) patients, therefore fulfilling AC diagnosis when this criterion was applied additionally to 1994 TFC, and thus fulfilling the new 2010 TFC. In 8 Group A patients (38%) a spontaneously occurring VT with LBBB morphology and superior axis has been recorded. In addition, this morphology was inducible with PES in 4 additional patients. In 4 Group A patients (19%) multiple VT morphologies were recorded during spontaneous episodes, and in 5 additional patients during PES. In total 16 of 21 Group A index patients did fulfil at least 1 of the new criteria.

In Group B, one family member had epsilon waves, and 8 had prolonged TAD. Because of both absence of spontaneous VT episode recordings and PES, VT criteria could not contribute to diagnosis in Group B patients. However, in total 7 Group B family members did fulfil new criteria.

## Summary

The 12-lead ECG is one of the most important tools for diagnosis of AC. In addition this tool contributes to evaluation of progression of the disease during follow-up. Since in AC ventricular arrhythmias and sudden death are due to re-entrant mechanisms, activation delay is a critical component. Recently a new parameter of activation delay, prolonged TAD, appeared to be superior in sensitivity compared to previously defined activation delay criteria, without loss of specificity. In addition, repolarization and new VT criteria contribute importantly to AC diagnosis.

### Conflict of interest statement

The authors declare that the research was conducted in the absence of any commercial or financial relationships that could be construed as a potential conflict of interest.
